# Application of Transcriptome-Based Gene Set Featurization for Machine Learning Model to Predict the Origin of Metastatic Cancer

**DOI:** 10.3390/cimb46070432

**Published:** 2024-07-09

**Authors:** Yeonuk Jeong, Jinah Chu, Juwon Kang, Seungjun Baek, Jae-Hak Lee, Dong-Sub Jung, Won-Woo Kim, Yi-Rang Kim, Jihoon Kang, In-Gu Do

**Affiliations:** 1 Oncocross Ltd., Seoul 04168, Republic of Koreawwkim@oncocross.com (W.-W.K.); 99yirang@oncocross.com (Y.-R.K.); 2 Department of Pathology, Kangbuk Samsung Hospital, Sungkyunkwan University School of Medicine, Seoul 03181, Republic of Korea; jinah.chu@samsung.com; 3 Yonsei Institute of Pharmaceutical Sciences, College of Pharmacy, Yonsei University, Incheon 21983, Republic of Korea

**Keywords:** cancer of unknown primary, metastatic cancer, machine learning, gene expression, transcriptome

## Abstract

Identifying the primary site of origin of metastatic cancer is vital for guiding treatment decisions, especially for patients with cancer of unknown primary (CUP). Despite advanced diagnostic techniques, CUP remains difficult to pinpoint and is responsible for a considerable number of cancer-related fatalities. Understanding its origin is crucial for effective management and potentially improving patient outcomes. This study introduces a machine learning framework, ONCOfind-AI, that leverages transcriptome-based gene set features to enhance the accuracy of predicting the origin of metastatic cancers. We demonstrate its potential to facilitate the integration of RNA sequencing and microarray data by using gene set scores for characterization of transcriptome profiles generated from different platforms. Integrating data from different platforms resulted in improved accuracy of machine learning models for predicting cancer origins. We validated our method using external data from clinical samples collected through the Kangbuk Samsung Medical Center and Gene Expression Omnibus. The external validation results demonstrate a top-1 accuracy ranging from 0.80 to 0.86, with a top-2 accuracy of 0.90. This study highlights that incorporating biological knowledge through curated gene sets can help to merge gene expression data from different platforms, thereby enhancing the compatibility needed to develop more effective machine learning prediction models.

## 1. Introduction

Finding the primary site of cancer is important for determining the treatment regimen for the cancer. Cancer of unknown primary (CUP) describes the diagnosis of metastatic cancer where the primary site of origin eludes detection despite comprehensive diagnostic evaluations [1]. CUP remains a perplexing challenge in oncology, representing approximately 3–5% of all malignancies a decade ago compared to 2–4% in recent years [1,2]. Nevertheless, CUP ranks as the third to fourth leading cause of cancer-related mortality [3]. CUP patients also exhibit greater levels of anxiety and depression than patients with known primary cancer and non-metastatic known primary cancer, along with impaired physical, mental, and social relationships [4]. Certainly, the majority of patients (80–90%) diagnosed with CUPs belong to an unfavorable group; their median overall survival (OS) spans from 3 to 11 months, with a 1-year OS of only 25–40% [5].

Identification of primary cancer site characteristics and associated expression targets enables the utilization of targeted anticancer drugs, enhancing prognosis over broad chemotherapy [3,6,7]. Ding et al. performed a meta-analysis, and determined that identifying the tumor of origin and administering targeted therapy are efficacious approaches, particularly for CUP patients with receptive tumor types [8]. However, traditional pathology methods, while considered the gold standard, often face limitations in exhaustively identifying the primary site due to tissue constraints and the complexity of diagnostic stains. Recent studies have demonstrated the potential of ML algorithms trained on diverse tumor and normal tissue datasets in discerning tissue-specific and tumor-specific patterns from high-resolution molecular data. Molecular diagnostic methodologies, exemplified by genome screening, have the potential to facilitate the identification of elusive origins in CUP cases [9,10]. Researchers are also using transcriptome-wide profiling to identify genes responsible for cancer or disease [11,12]. By utilizing gene expression data and employing sophisticated ML techniques, researchers have achieved notable successes in improving diagnostic accuracy for various cancers [13,14]. Using gene expression profiling (GEP) analysis, classical statistics and machine learning classification techniques can be used to predict primary cancers [15,16]. Researchers have used public transcriptome data to develop deep learning models that can identify genetic markers of primary and metastatic cancers, validating the model using newly acquired clinical samples [17,18]. SCOPE was trained on a collection of 10,688 untreated primary tumor samples and tested on 201 metastatic cancers. The model was validated on 15 cancer types, achieving an overall mean accuracy rate of 86% [17]. CUP-AI-Dx was trained on the transcriptome of 18,217 primary cancer samples, and external validation on metastatic cancer samples was performed on 92 metastatic cancer samples of 18 types collected from clinical laboratories in the US and Australia [18]. The resulting CUP-AI-Dx models showed top-1 accuracy results of 86.96% and 72.46%, respectively. Moon et al. developed OncoNPC, an XGBoost classifier based on next-generation sequencing, which identified distinct subgroups within CUP and improved treatment outcomes through genomically guided therapies [19]. OncoNPC was trained on 36,445 tumors from three medical centers and is able to classify 22 cancers; 971 CUP tumors were collected from the Dana-Farber Cancer Institute, and the model was able to identify the primary cancer type from metastatic carcinoma with 41.2% accuracy.

With the increase in the amount of tumor sample transcriptome data obtained through RNA sequencing, the amount of data available for learning is steadily increasing. However, a significant volume of cancer transcriptome data has been generated using micro-arrays, and compatibility must be ensured in order to utilize data from both platforms. Inter-platform compatibility can be achieved through featurization using gene set information that reflects biological knowledge [20]. By employing this approach, it is possible to construct a more extensive dataset for training, which is expected to improve accuracy. In this manner, our model was able to train more data than previous studies and make predictions for both micro-array and RNA sequencing data.

**Figure 1:**
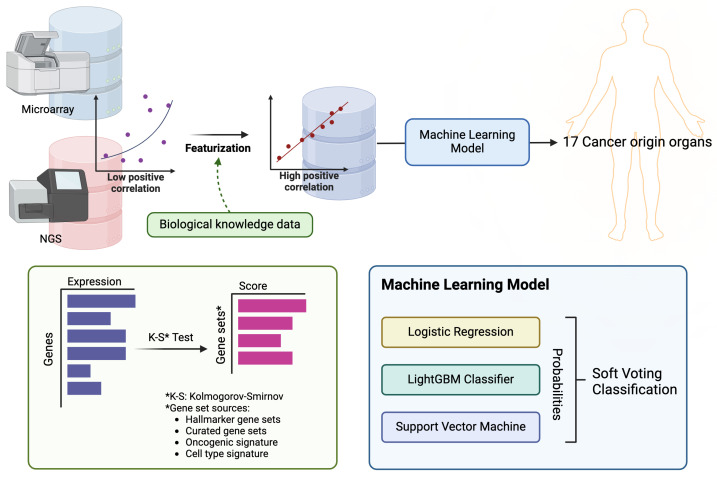
This figure presents the scheme of modeling process. RNA sequencing and micro-array data were collected from The Cancer Genome Atlas project (TCGA) and Oncopression (OCP), respectively, and featurization was conducted using gene sets representing the characteristics of each tissue and organ. Using the resulting feature scores, we developed a model to predict the primary site among 17 organs.

In this study, we used transcriptome data for 17 solid tumors from The Cancer Genome Atlas project (TCGA) [21] and Oncopression (OCP) datasets [22], and utilized training data featurized into gene set enrichment scores. We then trained a classification machine learning ensemble model by combining logistic regression, LightGBM, and SVM through a voting method (Figure 1). Furthermore, we conducted external validation using clinical samples collected from metastatic sites. Our ONCOfind-AI model showed a high robustness of 96.8 ± 2% with 5-fold validation on 27,941 primary cancer data samples. This is the largest number in studies that have built models from public data, and ONCOfind-AI produces the highest accuracy, with 86.2% for top-1 predicted sites and 90.0% for top-2 predicted sites on metastatic cancer samples in external validation.

## 2. Methods and Materials

### 2.1. Data Source

The data sources for training included transcriptome data for primary cancer collected from the OCP and TCGA databases. OCP data were created via micro-array with normalization [22], whereas the TCGA data, downloaded from Firehose (https://gdac.broadinstitute.org/, accessed on 1 July 2023), were created via RNA-Seq and normalized to RNA-Seq by expectation maximization (RSEM). A total of 27,941 samples for 17 cancer tissue type were selected for training (Table 1). For external validation, we used 103 formalin-fixed paraffin-embedded (FFPE) tissue samples from patients collected by Kangbuk Samsung Medical Center (KBSMC) between 2018 and 2022. Patient samples were collected after informed consent was obtained; the study followed the guidelines of the Declaration of Helsinki and received approval from the Institutional Review Board (KBSMC 2022-11-018). Additionally, public datasets comprising 107 samples across seven cohorts were obtained from the Gene Expression Omnibus (GEO) to further validate our findings (Table 2).

#### 2.1.1. RNA Sequencing and Gene Expression Profiling

mRNA was extracted from FFPE tissue samples taken from patients using an RNeasy FFPE Kit (Qiagen, Hilden, Germany) according to the manufacturer’s instruction. In summary, FFPE tissue sections were deparaffinized by treatment with deparaffinization solution and lysed by proteinase K digestion followed by heat treatment. Next, the supernatants treated with DNase were added to Buffer RBC and ethanol to adjust the binding conditions for RNA. The samples were applied to the RNeasy MinElute spin column, where the total RNA was bound to the membrane and contaminants were efficiently washed away. RNA was then eluted in RNase-free water. The RNA concentration was determined using a NanoDrop (Thermo Fisher Scientific, Waltham, MA, USA). Subsequently, RNA-seq was performed by Macrogen (Seoul, Republic of Korea) on the Illumina (San Diego, CA, USA) RNA-Seq platform for paired-end sequencing employing the SureSelectXT RNA Direct Reagent Kit (Agilent, Santa Clara, CA, USA). The raw FASTQ RNA-Seq data were trimmed using the Trimmomatic-0.39-1 tool [23]. Alignment and quantification were performed using STAR 2.7.8a and RSEM 1.3.3 with the GRCh38.105 genome reference [24,25].

### 2.2. Featurization and Feature Selection

**Figure 2:**
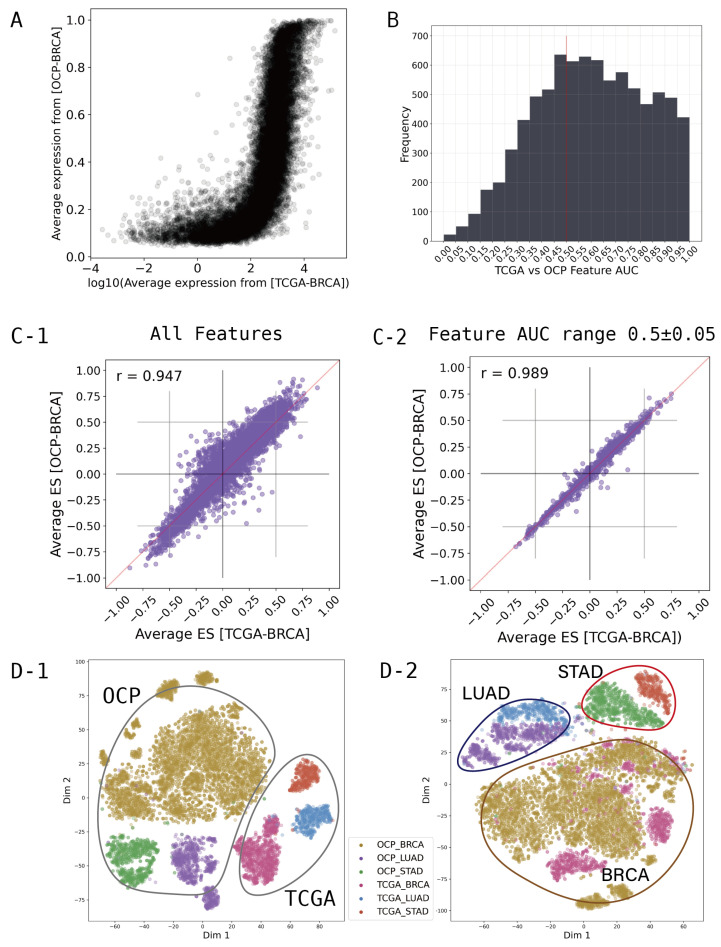
Featurization and feature selection: (**A**) average expression of each genes by groups from breast cancer (BRCA); (**B**) Histogram of TCGA-OCP distinguishing AUC value by gene sets. The red line marks 0.5; (**C**) Average enrichment score of each gene set by groups from BRCA; (**D**) Distribution of samples by T-SNE. (**C-1**,**D-1**) are when all 8300 gene sets are used, while (**C-2**,**D-2**) are when 1249 gene sets from AUC ranging from 0.45–0.55 are used.

Clinical data for external validation from KBSMC and GEO were quantified by transcriptome profile, as described in Section 2.1.1. Data from TCGA and OCP, the primary site cancer samples for training, are available in public databases and provide preprocessed transcriptome profiles. However, because the two public databases measure transcriptomes differently, they are normalized differently, and even samples from the same cancer type show different behavior. Figure 2A shows that the average expression levels of each gene between the TCGA and OCP groups for breast cancer are different in terms of both range and distribution pattern. To integrate the different characteristics of these data, we converted the gene-wise information of the transcriptome to the gene set dimension. We created gene set enrichment scores for all samples; statistical values were extracted using the Kolmogorov–Smirnov test with 8300 gene sets from the “Hallmarker gene sets”, “C2 curated gene sets”, “C6 oncogenic signature gene sets”, and “C8 cell type signature gene sets” obtained from MsigDB (www.gsea-msigdb.org, accessed on 5 March 2024, v2023.2) [26,27,28,29,30,31,32,33,34,35]. Positive or negative signs were assigned based on the directionality of the expression difference. Each gene set was defined by canonical pathway, cancer type, tissue type, cell type, and oncogenic gene. Therefore, we used the transcriptomic profiles collected from the tumors to calculate which gene sets are activated, allowing us to know which tissues, which tumors, and which pathways they originate from. To calculate the score per gene set, we used Gene Set Enrichment Analysis (GSEA) and calculated the normalized enrichment score for each gene set [26].

After computing the 8300 gene set scores, we investigated which of these could provide a comprehensive representation of the OCP and TCGA data. In feature selection, the receiver operating characteristic (ROC)-based feature selection approach can be used as an effective tool to evaluate individual features [36,37]. In particular, for binary-class problems the single feature classifier constructed from feature $f_{i}$ can establish an appropriate threshold θ. If x≥θ, then *x* is classified as the TCGA class, and if x<θ, then *x* is classified as the OCP class. If one feature $f_{i}$ has an area under the ROC curve (AUC) value for a single-feature classifier farther from 0.5 than another feature $f_{j}$, we can say that $f_{i}$ is more discriminative than $f_{j}$ in the two classes [36]. We calculated the AUC for each gene set feature in order to discriminate between the OCP cohort and TCGA cohort. Figure 2B shows a histogram of the AUC values calculated for each gene set feature. An AUC of 0.5 means that the classifier has no discriminative capacity at all, which means that OCP and TCGA can be used comprehensively when using features around 0.5. Therefore, feature selection was performed at intervals of ±0.05 around a baseline of AUC 0.5, and these selected feature groups were used for modeling.

We found that simply converting to gene set scores significantly normalized the ranges and distribution patterns of the OCP and TCGA cohorts. Figure 2(C-1)) shows all 8300 gene sets, demonstrating that the TCGA and OCP data are more highly correlated than at the gene level. Moreover, filtering gene sets that show differences between groups by AUC range can achieve a greater correlation (Figure 2(C-2)). When examining the distribution through T-SNE clustering for major cancer types such as lung, stomach, and breast cancer, compatibility between TCGA and OCP can be observed (Figure 2D). Dimension reduction was performed on the features using T-SNE from Scikit-learn in order to visualize the distribution of samples and groups in a two-dimensional space, further aiding in understanding and optimizing the model. Figure 2(D-1) is when all 8300 gene sets are used, while Figure 2(D-2) is when 1249 gene sets from the AUC range of 0.45–0.55 are used. In Figure 2(D-2), it can be seen that when using only the 1249 features selected by AUC, the features are more focused on the characteristics of the cancer type than those of the data type.

### 2.3. Cancer Primary Site Classification Model

To create a classification ensemble model based on machine learning, we used logistic regression, LightGBM, and support vector machine (SVM) with a soft voting classification approach. For this algorithm, we used the Scikit-learn (version 0.22.1) and LightGBM (version 3.1.1) Python packages. LightGBM is renowned for its high efficiency and low memory usage, which make it particularly effective for handling large datasets with high dimensionality. It also excels in terms of speed and accuracy. Additionally, SVM is advantageous for its effectiveness in high-dimensional spaces and its versatility through the use of different kernel functions, enabling it to model nonlinear boundaries. The ensemble approach combining these powerful classifiers leverages their individual strengths to enhance the predictive performance and achieve significant results. The parameters were set using a grid search.

### 2.4. External Validation

For external validation in metastatic cancer, predictions were made through the developed model using KBSMC and GEO data and a ranking was created based on the probability. To further validate the model under real-world conditions, we trained the model on public data and then evaluated it on a prospectively collected sample of cancer patients from the hospital. Through this process, the top-1 and top-2 prediction accuracy was calculated.

## 3. Results and Discussion

**Figure 3:**
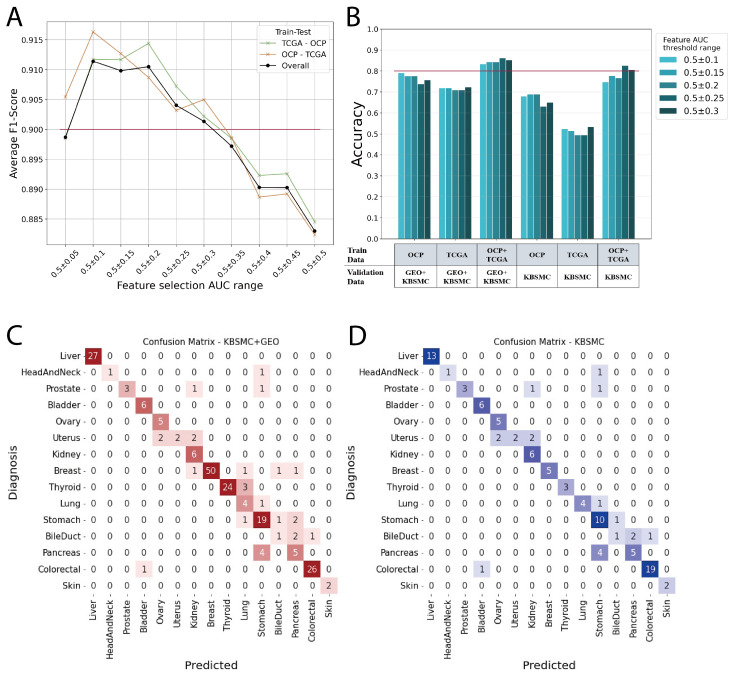
(**A**) TCGA vs OCP cross validation. Models were created to evaluate metastatic carcinoma using the AUC range of f1 scores of 0.9 and above, indicated by the red line; (**B**) Accuracy of external validation by feature selection groups; (**C**) Confusion matrix of external validation for KBSMC and GEO data; (**D**) Confusion matrix of external validation for only KBSMC data.

We demonstrate that more integrated data lead to better prediction performance on datasets consisting of real-world clinical data. In addition, we show how to integrate the two largest sources of transcriptomic data, namely, micro-arrays and RNA sequencing, and use them as features in a machine learning model. The model is found to be robust, with auccuracy scores of 0.988, 0.981, 0.965, 0.963, and 0.942 when performing a 5-fold cross-validation using randomized shuffling of the full TCGA + OCP data. Figure 3A shows the average F1 scores for each model with features in the range of AUC values shown in Figure 2B. In this case, the average F1 score is the average of the predictions across the 17 cancer primary organs. The model was trained with TCGA and then tested with cancer types from OCP data; conversely, the model was also trained with OCP data and then tested with TCGA cancer types. The overall line does not shown the median, as there is a larger amount of OCP data than TCGA data (19,541 vs. 8400) (Table 1). We found that AUC values of 0.5 ± 0.1 to 0.3 were associated with an average F1 score of 0.9 or higher (Figure 3A); therefore, we selected features from this range to build our model and conduct external data validation (Figure 3B).

Models trained on TCGA data, OCP data, and a combination of both TCGA and OCP were validated using external data. The best scores for the validation sets in each model are shown in Figure 3B and Table 3. Notably, models trained on TCGA data generated through RNA sequencing exhibited significantly lower performance when validated with KBSMC data, which were also produced through RNA sequencing. However, we found a significant improvement in performance when micro-array data from OCP were included in the training with our feature integration method. The models trained on the combined TCGA + OCP dataset demonstrated an accuracy range of approximately 0.80 to 0.86 depending on the range of feature selection (Figure 3B, Table 3). Figure 3C,D shows the confusion matrix for the top-1 prediction of the external validation set. The AUC of the feature selection group adopted in the evaluation model ranged from 0.5 ± 0.25. Most of the answers matched the actual primary site, and for those that were incorrect, the most likely answer was a neighboring organ. For example, when bile duct was the correct answer, the incorrect answers were pancreas and colorectum, whereas when the correct answer was uterus were the incorrect answers were ovary and kidney. In particular, the prostate, uterus, bile duct, and pancreas, which had relatively high incorrect answer rates, each had fewer than ten data points, making it difficult to perform sufficient validation. Our model had an average accuracy of 0.9 when calculating the accuracy for KBSMC+GEO data up to the top-2 predictions (Table 4).

### 3.1. Performance of ONCOfind-AI

We compared ONCOfind-AI to previous studies that have used machine learning to predict the primary site of metastatic carcinoma over the past five years (Table 5).

As it is very difficult to collect metastatic carcinomas, the models were all trained on cancer samples from the primary site. This study presents an integrated feature calculation method for the data types that provide transcriptome profiles, making it the largest study to use public data. The most recent study, OncoNPC from 2023, was a collaboration across three clinical research centers and collected the largest number of cancer samples; however, it achieved relatively low accuracy using a simple XGBoost classifier model. SCOPE used 201 metastatic cancer samples for validation, with 168 obtained from metastatic sites and the remaining 33 from the origin site. CUP-AI-Dx was developed at the Jackson Laboratory for Genomic Medicine and trained on RNA-Seq data from TCGA and the Cancer Genome Consortium (ICGC). For clinical validation, 92 FFPE samples representing 18 cancer types were collected from two clinical laboratories in the USA and Australia. Of these, 23 samples were from the JAX CLIA lab, six of of which were primary cancers. The primary site was predicted with 86.96% accuracy. The remaining 69 samples were from 18 types of metastatic cancer from the the University of Melbourne, for which the primary site was predicted with 72.46% accuracy. Although there is a large amount of primary cancer data, as there are many surgeries, commonly patients do not undergo surgery when metastasis occurs, making metastatic cancer data very rare and difficult to obtain. Therefore, most studies have only used a very small amount of testing data for clinical validation compared to the amount of training data. However, in our study all 210 samples were taken from metastatic tissues of metastatic cancer, and ONCOfind-AI predicted the primary site with the highest top-1 accuracy. Furthermore, as the answers that missed the top-1 prediction mentioned organs similar to the correct answer, it can be interpreted that our model learned the characteristics of tissues. This suggests that with more comprehensive feature selection based on the featurization approach outlined in our research, we can anticipate even better performance as the volume of data available across all cancer types increases.

### 3.2. Limitation and Future Work

This study shows the potential for further data integration by including other public databases such as ICGC, which we plan to address in the future. Collecting cancer samples from metastatic sites is very challenging, as is any study based on the resulting data, as patients with metastatic cancer often do not undergo biopsies. Our laboratory will continue to work with KBSMC to collect data and refine the model. In future research, we will validate whether our model could perform as well for other non-Asian ethnicities. Cancer genomic variation varies significantly by race, and only 672 out of 11,122 patients in the public TCGA database are Asian [21]; therefore, we expect our model to perform better for Americans.

## Figures and Tables

**Table 1:** Training datasets.

Cancer Type	Data Source	Sample No.
Adrenal Gland	OCP	311
	TCGA	261
Bile Duct	OCP	184
	TCGA	36
Bladder	OCP	310
	TCGA	408
Brain	OCP	3054
	TCGA	696
Breast	OCP	5543
	TCGA	1093
Colorectal	OCP	3074
	TCGA	381
Head And Neck	OCP	622
	TCGA	520
Kidney	OCP	356
	TCGA	891
Liver	OCP	413
	TCGA	373
Lung	OCP	2243
	TCGA	1018
Ovary	OCP	1143
	TCGA	307
Pancreas	OCP	207
	TCGA	178
Prostate	OCP	247
	TCGA	497
Skin	OCP	294
	TCGA	103
Stomach	OCP	920
	TCGA	599
Thyroid	OCP	298
	TCGA	501
Uterus	OCP	322
	TCGA	538
**Total**	**OCP**	**19,541**
	**TCGA**	**8400**
	**OCP + TCGA**	**27,941**

**Table 2:** Validation datasets.

Cancer	Data Source	Sample No.
Bile Duct	KBSMC	4
Bladder	KBSMC	6
Breast	GSE14017	29
	GSE147995	13
	GSE191230	7
	KBSMC	5
Colorectal	KBSMC	20
	GSE40367	7
Head And Neck	KBSMC	2
Kidney	KBSMC	6
Liver	GSE40367	15
	KBSMC	13
Lung	KBSMC	5
Ovary	KBSMC	5
Pancreas	KBSMC	10
Prostate	KBSMC	5
Skin	KBSMC	2
Stomach	KBSMC	11
	GSE246963	8
	GSE191139	4
Thyroid	GSE60542	24
	KBSMC	3
Uterus	KBSMC	6
**Total**	**KBSMC**	**103**
	**GEO**	**107**
	**Total**	**210**

**Table 3:** External validation results.

Training Data	Test Data	Feature AUC Range 0.5±	Weighted Accuracy
OCP + TCGA	GEO + KBSMC	0.25	0.862
OCP + TCGA	GEO + KBSMC	0.3	0.852
OCP + TCGA	GEO + KBSMC	0.15	0.843
OCP + TCGA	GEO + KBSMC	0.2	0.843
OCP + TCGA	GEO + KBSMC	0.1	0.833
OCP + TCGA	KBSMC	0.25	0.825
OCP + TCGA	KBSMC	0.3	0.806
OCP	GEO + KBSMC	0.1	0.79
OCP + TCGA	KBSMC	0.15	0.777
OCP	GEO + KBSMC	0.2	0.776
OCP	GEO + KBSMC	0.15	0.776
OCP + TCGA	KBSMC	0.2	0.767
OCP	GEO + KBSMC	0.3	0.757
OCP + TCGA	KBSMC	0.1	0.748
OCP	GEO + KBSMC	0.25	0.738
TCGA	GEO + KBSMC	0.3	0.724
TCGA	GEO + KBSMC	0.15	0.719
TCGA	GEO + KBSMC	0.1	0.719
TCGA	GEO + KBSMC	0.2	0.71
TCGA	GEO + KBSMC	0.25	0.71
OCP	KBSMC	0.15	0.689
OCP	KBSMC	0.2	0.689
OCP	KBSMC	0.1	0.68
OCP	KBSMC	0.3	0.65
OCP	KBSMC	0.25	0.631
TCGA	KBSMC	0.3	0.534
TCGA	KBSMC	0.1	0.524
TCGA	KBSMC	0.15	0.515
TCGA	KBSMC	0.25	0.495
TCGA	KBSMC	0.2	0.495

**Table 4:** Top-2 accuracy of external validation.

Cancer Type	Top-2 Accuracy for GEO + KBSMC Validation
BileDuct	0.250
Bladder	1.000
Breast	0.926
Colorectal	0.963
HeadAndNeck	0.500
Kidney	1.000
Liver	0.964
Lung	0.800
Ovary	1.000
Pancreas	0.900
Prostate	0.800
Skin	1.000
Stomach	0.870
Thyroid	0.889
Uterus	0.667
**Weighted Average**	0.900

**Table 5:** Performance comparison.

Model	Train Set	Test Set (Metastatic)	Average Accuracy
SCOPE [17]	10,688	201 (33 from origin site)	15 types, 86%
CUP-AI-Dx [18]	18,217	92 (23 from origin site)	18 types, 83.33%
OncoNPC [19]	36,445	971	22 types, 41.2%
**ONCOfind-AI**	**27,941**	**210**	**17 types, 86.2%**

## Data Availability

The data that support the findings of this study are available on request from the corresponding author, In-Gu Do. The data are not publicly available due to their containing information that could compromise the privacy of research participants. The source code for this project has been distributed on GitHub (http://github.com/yeonuk-Jeong/ONCOfind, accessed on 2 July 2024).
